# Distinguishing Mechanisms for Reactive Uptake at Liquid Surfaces via Angular Distributions of Inelastically Scattered Molecules

**DOI:** 10.1021/acs.jpca.4c02917

**Published:** 2024-06-25

**Authors:** Maksymilian J. Roman, Adam G. Knight, Daniel R. Moon, Paul D. Lane, Matthew L. Costen, Kenneth G. McKendrick

**Affiliations:** Institute of Chemical Sciences, Heriot-Watt University, Edinburgh EH14 4AS, U.K.

## Abstract

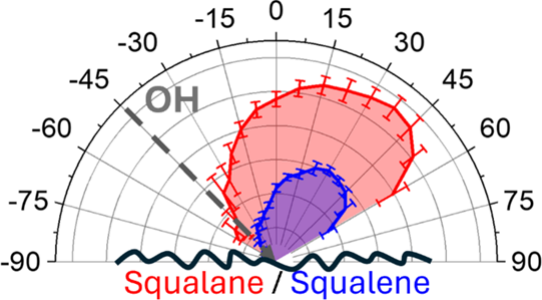

Angular distributions of OH inelastically scattered from the surfaces of the reactive hydrocarbon liquids squalane (fully saturated) and squalene (partially unsaturated) have been measured. A pulsed, rotationally cold molecular beam (*E*_i_ = 35 kJ mol^–1^) of OH was scattered from refreshed liquid surfaces in a vacuum. Spatially and temporally resolved OH number densities were measured by pulsed, planar laser-induced fluorescence. Results are compared with those for the inert liquid perfluoropolyether. The clearly asymmetric distributions for 45° incidence add to the weight of evidence for predominantly impulsive scattering from all three liquids. However, we propose that significant differences in their shapes may be diagnostic of contrasting reaction mechanisms. Direct, near-specular trajectories survive preferentially on squalene, consistent with an addition mechanism removing those at more backward angles. This trend is reversed for squalane, as expected for direct abstraction. The results reinforce the need to consider the effects of composition-dependent contributions from different reaction mechanisms in the modeling of OH-aging of atmospheric aerosol particles.

## Introduction

Reactions at the gas–liquid interface play an important role in many processes of practical interest, spanning, for diverse examples, respiration in human lungs to industrial applications of multiphase catalysis. Despite this significance, it is generally the case that elementary processes at liquid surfaces have been much less studied than homogeneous reactions in the gas phase or their counterparts at gas–solid surfaces. We explore here new dynamical aspects of reactive collisions of OH radicals at liquid surfaces chosen for their relevance in the chemistry of atmospheric aerosol particles.

Organic atmospheric aerosols result from both natural and anthropogenic sources. Examples of primary aerosols include those in sea spray, which are rich in organic surfactants from the sea-surface microlayer,^1^ and the dispersion of disintegrated plant materials by the wind which produces supermicron wax particles consisting of large *n*-alkane compounds.^2^ Secondary organic aerosols are produced indirectly following the oxidation of precursor emissions of volatile or semivolatile organic compounds by ambient species, including the OH radical, resulting in a less-volatile organic product that can nucleate and precipitate out of the gas phase.^3^ Once formed, the organic surfaces of aerosol particles can undergo further oxidation through heterogeneous reactions with atmospheric OH in a process referred to as “aging”.^4^ This is the aspect of aerosol chemistry to which the work reported here is the most relevant. Its practical implications are substantial because it alters the light-scattering, light-absorption, and cloud-formation properties of the aerosols. These changes, in turn, affect air quality, visibility, climate, and even human health.^5,6^ It is therefore clearly desirable to better understand the interactions between gaseous OH and organic aerosol surfaces to allow these environmental impacts to be modeled and predicted reliably.

As is obvious from the diversity of sources and the secondary modification that they undergo, organic aerosol surfaces can be very complex. This makes it very challenging to gain insight into elementary reaction mechanisms for the different functionalities present on the surface through experiments on real aerosol particles. It is, therefore, common practice, as we do here, to study proxy surfaces of pure liquids representing the types of functionalities likely to be present in aerosol particles. In particular, the long-chain branched hydrocarbons squalane (C_30_H_62_; 2,6,10,15,19,23-hexamethyltetracosane) and squalene (C_30_H_50_; 2,6,10,15,19,23-hexamethyl-2,6,10,14,18,22-tetracosahexaene) have been extensively used as proxy saturated and unsaturated surfaces, respectively, in experimental studies of oxidation by OH and other relevant species.

Most experiments in this field have typically used flow-tube or continuous-flow stirred-tank methods to measure the reactive uptake of OH. The model hydrocarbons are present as either bulk surfaces or, less commonly, small particles.^7−9^ They have provided most of the available information on overall uptake coefficients, which may include contributions from secondary reactions and important insights into other phenomenological aspects of the kinetics including end-product speciation, changes of oxidation state, and material loss from the surface during oxidation. However, by design, they do not make it easy to isolate the contributions from individual elementary processes in the mechanism.

A series of fundamental molecular-scattering studies by our own group have sought to understand the dynamics of the primary OH collisions with hydrocarbon surfaces in more detail. These studies form part of a wider recent surge of interest in elementary gas–liquid scattering.^10−13^ Broadly speaking, most work in this field has combined either molecular-beam or photolytic sources of projectiles with continually refreshed surfaces formed using the well-established rotating-wheel technique.^14^ The scattered products have been detected by either mass spectrometry or some form of optical spectroscopy. It has been common to frame the discussion of the results of such studies in terms of impulsive scattering (IS) and thermal desorption (TD) mechanisms, introduced originally in the related field of gas–solid scattering.^10−13^ In IS, the projectile suffers one collision, or at most a small number of collisions, with the surface. Its resulting speed, angular, and (if molecular) internal-state distributions are dynamically determined and related to those of the incident molecule. In contrast, for TD, the molecule has interacted sufficiently strongly with the surface that it has lost all memory of the initial conditions and leaves the surface with characteristics of a Maxwell–Boltzmann distribution at the surface temperature and a cosine angular distribution about the surface normal (“Knudsen’s law”). It is important to stress that there is considerable evidence that these are only approximate, limiting cases whose boundaries are blurred at the atomic level for real systems, but nevertheless, they are empirically useful for characterizing experimental observations.^12^

In our own work on OH, laser-induced fluorescence (LIF) was used to detect the incident and scattered molecules.^15−18^ The results highlighted the dependence of the reactive uptake of OH on the collision energy and surface structure. Recently, we introduced a novel planar-LIF (pLIF) imaging technique, extending the dynamical information that can be extracted.^19−21^ The first such study established, through rotational-state specific measurements of superthermal scattered speeds, that OH scattered from surfaces of inert perfluoropolyether (PFPE), squalane, and squalene via a predominant IS mechanism.^19^ There was little evidence of a significant TD component at the relevant modestly superthermal (*E*_i_ = ∼35 kJ mol^–1^) collision energies. This dominance of IS under these conditions has most recently been further confirmed for OH inelastic scattering from PFPE in the first study to successfully resolve the state-specific angular scattering distributions and their correlations with scattered speed.^20^ The reliability of the measured distributions was confirmed in a companion paper describing extensive numerical modeling of geometrical and other effects.^21^

The work here is an extension of these imaging experiments in which rotational-state-dependent angular distributions are measured successfully for OH inelastically scattered from *reactive* liquids, squalane and squalene, for the first time. Our main new proposition is that it may be possible to infer information about reaction mechanisms from the angular distributions of those OH molecules that survive a collision with the surface, i.e., which molecules are “missing” because they have reacted.

## Experimental Methods

The experimental method has been described in full detail elsewhere.^20^ In essence, a short packet of OH was created via a pulsed, high-voltage (HV) discharge in a mixture of H_2_O in He. The gas expanded supersonically to form a molecular beam (MB) that was rotationally cold (a two-temperature fit gives *T*_rot,1_ = 57 K, *T*_rot,2_ = 164 K; α = 0.43, where α is the proportion of the population with *T*_rot,1_) but translationally superthermal (most-probable laboratory-frame kinetic energy, *E*_i_ = 35 kJ mol^–1^; most-probable speed, *v*_peak_ = 2045 ± 35 m s^–1^, as determined in auxiliary measurements by changing the source-to-observation distance^20^).

This beam was directed at continually refreshed room-temperature liquid surfaces, formed in vacuo using the rotating-wheel technique.^14^ Squalane (Sigma-Aldrich, 99% purity) and squalene (Sigma-Aldrich, ≥ 98% purity) were used without further purification other than the removal of air, water, and any other dissolved volatiles that naturally occur when they are placed under vacuum. In this work, incidence angles, θ_i_, of 0 and 45° were used.

The spatial distribution of OH in specific rotational states, *N′*, was measured by pLIF, excited by a thin (∼4 mm thick × ∼30 mm wide) sheet of pulsed laser light. The laser sheet propagated in the scattering plane, containing the incident beam and the normal to the surface of the liquid, with its closer edge at a short distance (∼10 mm) in front of the liquid surface. OH LIF was captured with retention of its spatial distribution by an imaging assembly (consisting of a telescope, dichroic filter to isolate OH emission on the A-X(1,1) band, dual-MCP imaging intensifier, and camera) positioned directly above the probe region. OH was probed in separate experiments in three levels (*N′* = 2, 3, 4) of the lower ^2^Π_3/2_ manifold of *v*′ = 0 via Q_1_-branch transitions of the A-X(1,0) band; scattering into *N′* = 1 could not be measured reliably because of the dominant contribution from *N′* = 1 in the incident beam, as explained previously.^20^

Two distinct types of data were collected. *Image sequences* were obtained by averaging the images from a moderate number (500) of single laser shots at a series of closely spaced delays between the pulsed HV discharge and the probe-laser pulse. As explained previously, these are more suitable for determining the most-probable scattered OH speeds.^20^ Image sequences were recorded for θ_i_ = 0 and 45°. *Extended images* were the accumulation of images from a much larger number of laser shots (50,000) at fixed delays.^20^ They were recorded to quantify the angular distributions for θ_i_ = 45° more precisely, as explained below.

Contributions from the incident beam were removed from both types of data by subtracting the corresponding image taken at the same delay without a liquid surface being present. This subtraction also removed a low and essentially uniform background due to the electronic noise in the camera. In the case of squalane, the images required additional processing to remove an unexpected contribution from fluorescence of the liquid on the wheel exposed to scattered probe light. (This must presumably result from a minor impurity in the squalane, which does not itself absorb at these wavelengths.) This process is described in the Supporting Information (Section S1). The regions affected lay outside the main region used in the analysis.

In the subsequent analysis of images, *regions of interest* (ROIs) were delimited by a series of concentric arcs divided by radii at a sequence of angles, in conceptually the same way as in previous work.^19−21^ In practice, the arcs were centered at distances of 14, 20, 26, 31, and 37 mm, measured to the midpoints of the ROIs from the central point of impact of the MB on the surface. The radii spanned final scattering angles, θ_f_, from −67.5 to +67.5° in 7.5° steps. (Negative angles, by convention, indicate scattering to the incident (left-hand in figures below) side of the normal to the surface when θ_i_ = 45°.)

The process by which the intensities are corrected for the instrument function (IF correction) has been described in detail elsewhere.^20,21^ The IF is the result of the combined variations in intensity across the probe-laser sheet (perpendicular to the propagation direction); the efficiency with which LIF is collected and transmitted from different spatial positions by the imaging system; and any spatial variations in the gain of the image intensifier or sensitivity of the camera. In brief, the IF was measured in separate experiments by flooding the chamber with OH using a much longer HV discharge pulse and with a longer discharge-probe delay, allowing the OH to thermalize before recording the pLIF image. We assume that this corresponds to a uniform distribution of the OH density in the probe region. Correction for the IF was achieved by dividing a scattering image by a smoothed version of this uniform-density IF image, confined to regions where the intensity in the IF image was above a chosen threshold.

## Results

### Speed Distributions from Image Sequences

**Figure 1:**
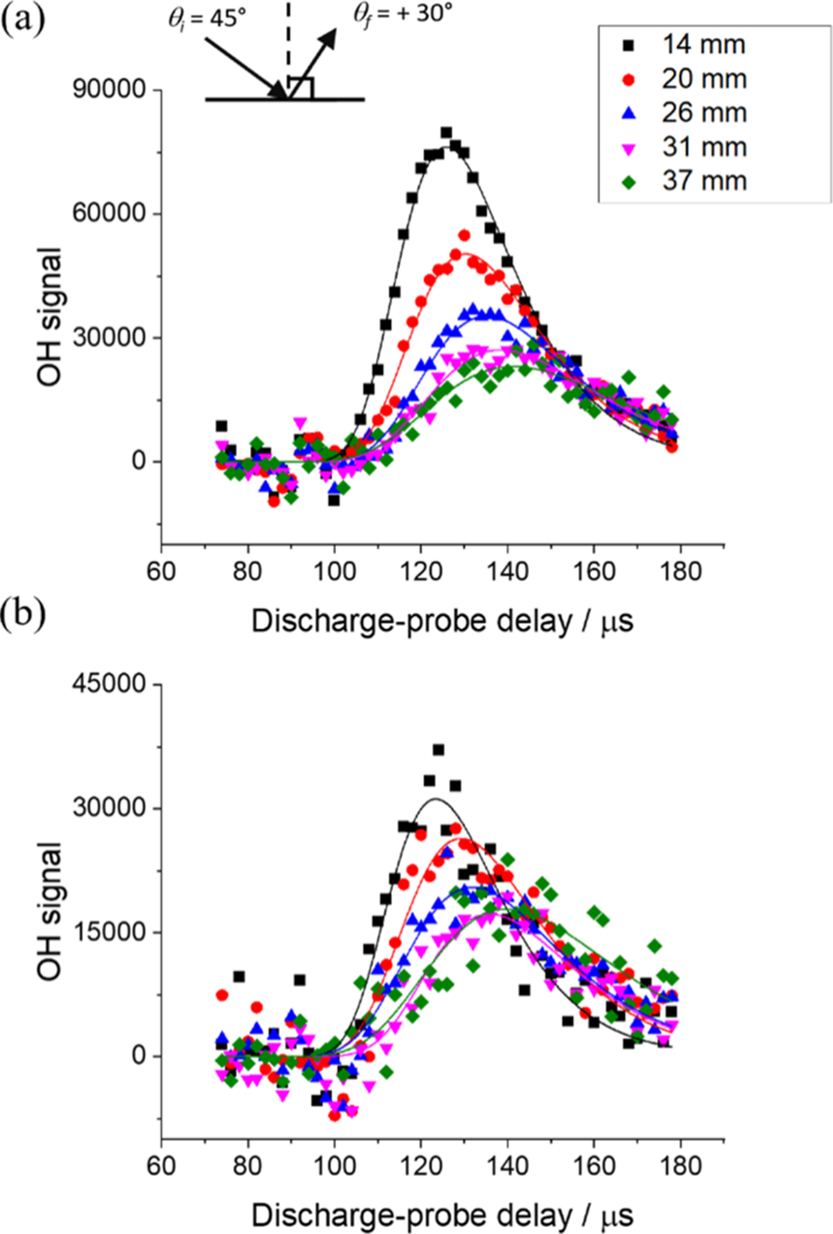
TOF profiles for OH *N*′ = 2 scattered from (a) squalane and (b) squalene, with θ_i_ = 45° and θ_f_ = +30° (see inset). Profiles shown are for ROIs at 5 different distances from the surface (14, 20, 26, 31, and 37 mm; black, red, blue, magenta, and green, respectively). Solid lines are Gumbel distribution fits (same color coding).

As described in detail in our previous work,^19,20^ the most-probable speeds are derived by analyzing the propagation of the scattered wave of OH across the probe region during an image sequence. In practice, the time-of-flight (TOF) profiles across a series of RoIs that extend along a fixed value of θ_f_ are fitted to an arbitrary function (the Gumbel distribution^20^ is found to be suitable), from which the delay corresponding to the peak is extracted. Examples are shown in Figure 1 for scattering into *N*′ = 2 with θ_i_ = 45° and θ_f_ = +30° from squalane and squalene. The most probable speed is the inverse of the slope of a plot of measured peak delay against the known RoI radial distance.

Overall, the measured most-probable speeds broadly confirm the trends in our previous proof-of-concept work,^19^ which we still believe to be qualitatively reliable (but subject to a quantitative correction due to improved calibration of the absolute distance scale).^20,21^ We therefore summarize them only briefly here. The average most probable speeds over all final angles, weighted by the known relative populations of the observed rotational levels *N*′ = 2–4, are given in Table 1 for both incidence angles. The full results for squalane and squalene from which these averages are derived are given in the Supporting Information (Section S2).

**Table 1:** Most-Probable Speeds, Averaged over All Final Angles and Weighted by the Relative Populations of the Observed Rotational Levels *N*′ = 2–4, for θ_i_ = 0 and 45°^a^

liquid surface	weighted-average most-probable speed/m s^–1^	
	θ_i_ = 0°	θ_i_ = 45°
squalane	1180 ± 60	1340 ± 100
squalene	1250 ± 90	1400 ± 120
PFPE^b^	1470 ± 120	1590 ± 180

a The errors are 1σ standard errors that are also weighted by relative populations.

b As reported in ref (20).

The most-probable scattered OH speeds from both squalane and squalene are clearly significantly superthermal, regardless of incidence and final angles; for comparison, the most-probable speed in an OH thermal distribution at 300 K is 540 ms^–1^. They show only marginal differences between squalane and squalene within the estimated errors (see Tables S1 and S2 in Supporting Information). For θ_i_ = 0°, any variations with θ_f_ are within the uncertainties. For θ_i_ = 45°, there is a systematic, approximately linear, modest increase in speed from negative to positive θ_f_ for both liquids, similar to those observed previously.^19^ These trends with incidence and scattering angles are similar to those from PFPE, but the absolute speeds are systematically slower.^20^

### Angular Distributions from Image Sequences

The angular distributions were extracted from the image sequences, to which the IF correction had been applied, by integration of the TOF profiles through selected RoIs between the chosen limits. For each product rotational state, the three innermost arcs of RoIs were analyzed separately, and the final results averaged as explained below. To investigate whether the angular distribution changed significantly during the profile, two intervals were chosen: 110–132 μs, which corresponds to the rising edge up to the approximate peak of the profile; and 152–170 μs, which overlaps the tail.

Two corrections were applied to these raw integrals to convert them to relative fluxes as a function of θ_f_. (The effects of both corrections will be illustrated more explicitly below for the more refined angular distributions from the extended images.) The first is the finite-beam (FB) correction, which, as we have explained in detail previously,^20^ accounts for the effect of the non-negligible MB diameter on the observed angular distribution. We have derived quantitative correction factors, which are specific to the radial distance of each arc from the surface, from extensive Monte Carlo (MC) simulations of the experimental geometry.^21^

The second correction is a new development in this work, which we have derived through further MC simulations to quantify any potential differences in the observed distributions for scattering from different liquids due to flux-density effects; we term this the FD correction. Further details are given in the Supporting Information (Section S3). In essence, the FD correction accounts for differences in scattered speeds as a function of θ_f_. This variation has a negligible effect in all cases for θ_i_ = 0°. However, it is non-negligible for θ_i_ = 45°, for which the MC modeling shows that there is a typically ∼20% variation in observed number density for a fixed flux between the slowest, most backward (θ_f_ = −60°) and fastest, most forward (θ_f_ = +60°) scattering angles. Importantly, though, the differences in this correction between the three liquids squalane, squalene, and PFPE (for which we have applied the result of this new analysis here to the published data^20^) are marginal. This is because although there are systematic differences in absolute speed between liquids (see Table 1), the relative variation in speed with θ_f_ is similar for all three liquids (see Supporting Information, Sections S2 and S3).

**Figure 2:**
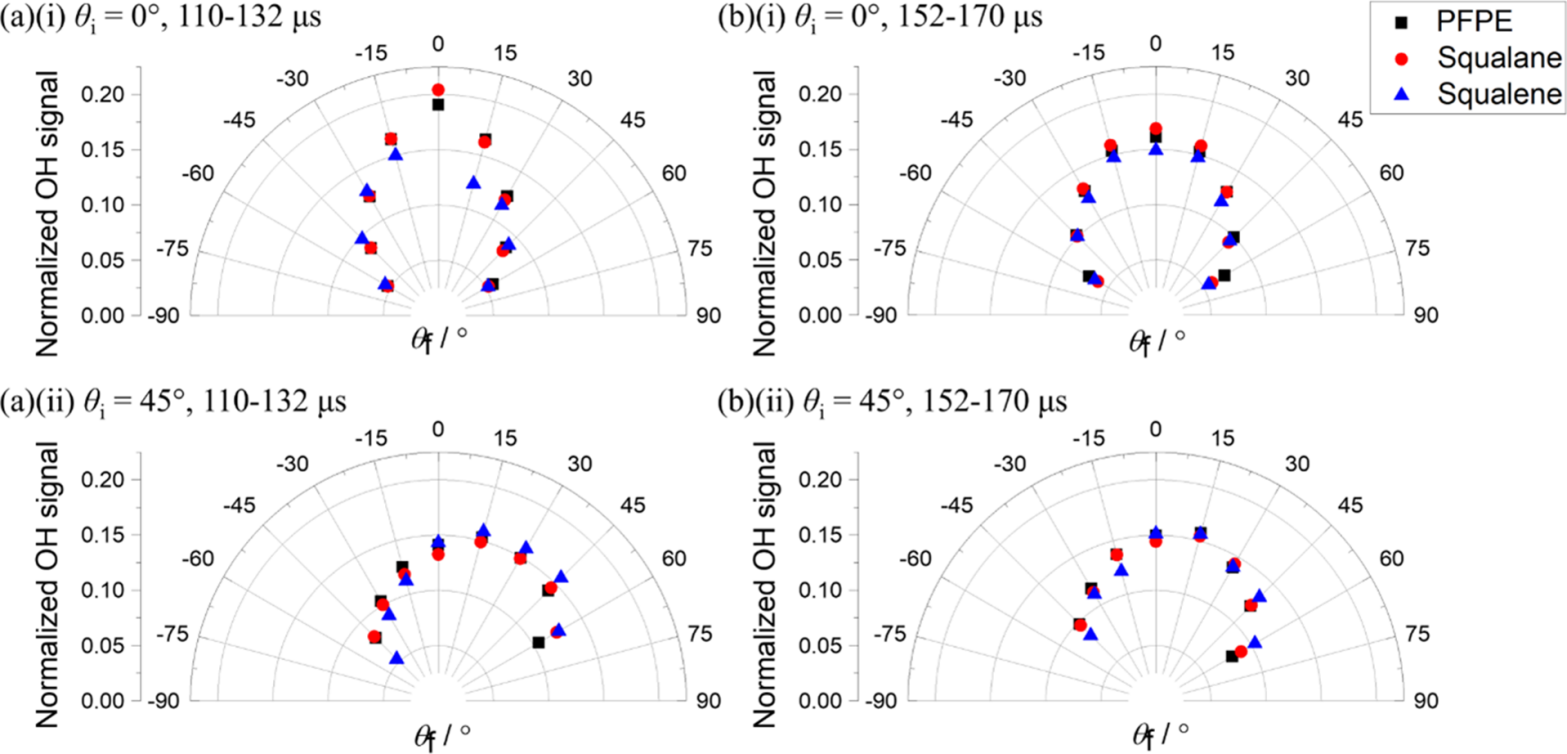
Relative OH fluxes, normalized over the range final angles (−60° < θ_f_ < +60°) for which they were collected, from image sequences for squalane (red circles) and squalene (blue triangles), compared with PFPE (black squares) from data in ref (14). Incidence angles (i) θ_i_ = 0° and (ii) θ_i_ = 45°. Results are a weighted average over OH *N*′ *=* 2–4, averaged over the three innermost RoIs along each θ_f_. Relative fluxes were derived by applying the FD correction (see text) to integrated number densities in the delay ranges indicated (a) 110–132 and (b) 152–170 μs. θ_i_-dependent corrections for the finite size of the incident MB (FB correction—see text) have also been applied. No result is shown for squalene in (a)(i) because of unreliable subtraction of the incident beam from the relatively small scattered signal. The remaining points have been renormalized appropriately; those at θ_i_ = ±15° may also be affected.

The fully corrected flux angular distributions from the image sequences for all three liquids are shown in Figure 2. The data shown are averages of the results for OH *N′* = 2–4, weighted by their known relative populations in the rotational distributions from each liquid (see Supporting Information, Section S4).^18^

Some features and trends are immediately obvious in Figure 2. For θ_i_ = 0°, the distributions are more sharply directed back along θ_f_ = 0° at shorter delays. They broaden at later times but are still narrower than a cos θ_f_ distribution (which would be circular in this construction). Any differences between liquids are marginal. (Note that no result is reported for θ_f_ = 0° with θ_i_ = 0° for squalene; this is the most difficult case for subtraction of the incident beam from the scattered signal, which could not be achieved reliably due to the low OH survival probability for squalene.)

For θ_i_ = 45°, the distributions are clearly extremely different from those for θ_i_ = 0°. They are obviously asymmetric about the surface normal. This has been reported previously for PFPE,^20^ but it is the first time it has been observed for squalane or squalene, the angular resolution having been insufficient in our earlier proof-of-concept work.^19^ The distribution broadens, with a shift in the most probable angle to lower θ_f_, at later delays, but some asymmetry clearly persists. There are now indications of subtle differences between liquids, with squalene producing a slightly sharper angular distribution that is more noticeable during the earlier delays.

These preliminary observations motivated the more incisive investigation of the angular distributions below using extended images which are better-adapted to determining them.^20^

### Angular Distributions from Extended Images

**Figure 3:**
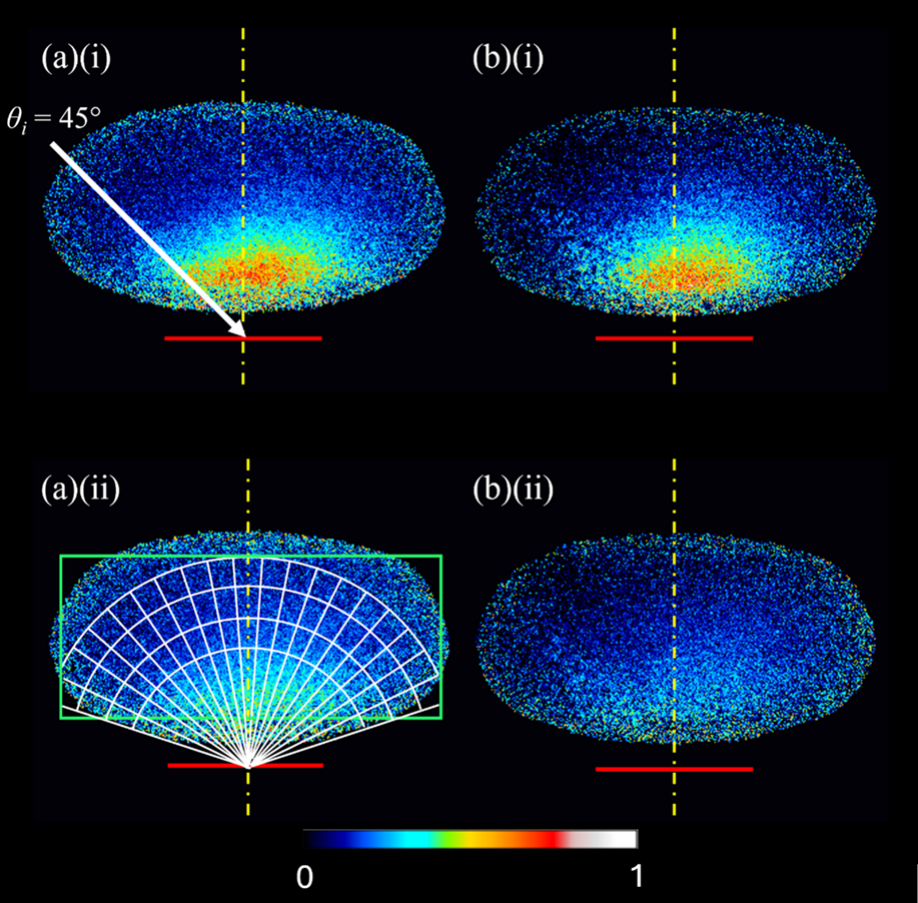
Extended images showing (a) OH (*N*′ = 3) scattered from squalane and (b) OH (*N*′ = 2) scattered from squalene at (i) 132 and (ii) 152 μs after the creation of OH in the discharge. Any contributions from residual incident beam and other background signals have been subtracted (see Experimental Methods). The pixels were false-colored to indicate their intensities; black represents no intensity and white is the maximum intensity. A relative intensity scale is located at the bottom of the figure. The elliptical shape of the images results from correcting the pixel intensities with the IF (see Experimental Methods). The intensity scale of the (b) images was adjusted by the ratio of survival probability of OH on squalane and squalene (ca. 0.7/0.3) to allow for an easy visual comparison of OH signals. The relative intensities between parts (i) and (ii) reflect their measured intensities for both parts (a) and (b). The white arrow in (a)(i) shows the 45° incidence angle of the OH beam. The positions of the liquid surface (red solid line) and the normal to the surface at the center of the area dosed by the ingoing MB (yellow dashed line) are indicated. The white arcs and lines in (a)(ii) define the ROIs used during the analysis, whereas the green rectangle delimits the analysis region.

We concentrate on θ_i_ = 45° for the extended images because they are less prone to problems with subtraction of the incident beam and in any case, as suggested by Figure 2, are dynamically more insightful due to the breaking of cylindrical symmetry around the surface normal. Example extended images for scattering with θ_i_ = 45° from each of squalane and squalene at delays of 132 and 152 μs are shown in Figure 3. These delays correspond to the peak of the scattered OH signal and then later by approximately the temporal width (fwhm = ∼20 μs) of the incident OH packet, respectively. They are chosen to represent the majority, faster scattered OH and the slower tail, respectively. The data shown are for OH in the *N′* = 2 (squalene) and *N′* = 3 (squalane) rotational levels, chosen to give more easily comparable signal sizes at the earlier delay. The relative intensities at the different delays for each liquid correctly reflect the measured signal sizes: we consider the significance of the clear differences in these ratios for squalane (i.e., (a)(i) versus (a)(ii)) and for squalene ((b)(i) versus (b)(ii)) below. Similar data were obtained for the other *N′* levels for each liquid. The location of the surface, identified precisely in auxiliary measurements,^20^ and the approximate dimensions of the area probed by the laser sheet are indicated. The images have been corrected in a pixelwise fashion for variations in the detection sensitivity (i.e., the IF correction), via the procedure described above (see Experimental Methods) and in more detail elsewhere.^20^ Other signals, including any residual ingoing beam, were removed by subtraction. The intensities in Figure 3 therefore reflect the spatial distribution of the scattered OH number density.

Even without further processing, the primary feature of the results from the θ_i_ = 45° image sequences in Figure 2 can be confirmed by eye in Figure 3. For both liquids at both delays (and all rotational levels, including those not shown explicitly here), the angular distribution is asymmetric with respect to the surface normal, with more OH on the specular side.

Quantitative angular distributions were extracted by first summing the pixel intensities in each RoI in the IF-corrected images using the pattern of ROIs described above and shown schematically in Figure 3a(ii). We once again applied the FB correction to account for the non-negligible width of the MB to convert these to undistorted distributions of integral number density as a function of scattering angle, θ_f_.^21^ There is a slight subtlety here in that the extended images produce a snapshot of positions at a fixed delay as opposed to the integral over a finite interval that was used in the analysis of the image sequences. However, further MC modeling showed that any quantitative differences in the FB corrections required in these two scenarios were insignificant (see Supporting Information, Section S5). Finally, we applied the FD correction to convert from measured number density to relative flux, which we assume to apply equally to the extended images (see Supporting Information, Section S3). The results were averaged over three inner arcs of ROIs.

The full set of angular distributions for each rotational level and delay for both liquids (and, for comparison, PFPE, based on reanalysis of the previous data^20^) are provided in the Supporting Information (Section S6). The relatively modest effects of each of the FB and FD corrections, which fortuitously partially cancel for understandable reasons described in the Supporting Information, are shown explicitly there. There are some detailed differences between the rotational levels for a given liquid. There may be some evidence for a shift toward more subspecular (i.e., peaking closer to the surface normal) scattering for the highest level, *N*′ = 4, seen previously for PFPE and for which possible dynamical explanations were proposed.^20^ However, the trends between individual levels are less clear for squalane and squalene because the measurements are more challenging than those for PFPE due to the reduction in OH survival probability and hence lower signal-to-noise ratios, so we do not attempt to draw detailed conclusions from them here.

**Figure 4:**
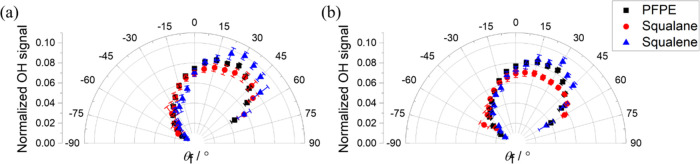
Peak-normalized angular distributions of OH scattered with θ_i_ = 45° from PFPE (black squares, from a reanalysis of data in ref (14), squalane (red circles), and squalene (blue triangles) taken (a) 132 μs and (b) 152 μs after the creation of OH in the discharge. The distributions were weight-averaged for the relative populations of the three probed OH rotational levels (*N′* = 2, 3, and 4). Some points subject to exceptionally large subtraction errors in the vicinity of the incident beam have been omitted.

The feature that we wish to highlight is the distinct and reproducible difference between the overall form of the angular distributions for squalane and squalene. This is illustrated in Figure 4, where an average has been taken over the distributions for *N*′ = 2–4, weighted by the relative integral populations of the levels scattered from each liquid. (See Supporting Information, Section S4, for details of the weighting procedure; we are characterizing the mechanical rotational distributions via the Q_1_ branches, which monitor one Λ- doublet in the majority F_1_ manifold, but do not expect significant differences between Λ- doublets or substantially different angular distributions in the minority F_2_ manifold. The omission of *N′* = 1 from these averages is not expected to make a significant material difference; similarly, the fraction of the population in higher unobserved levels with *N*′ ≥ 5 is small and similar for all liquids, and hence, its inclusion would be unlikely to introduce significant differences in the weighted averages for different liquids.) We also include the equivalent reanalyzed results for PFPE.^20^ Uncertainties shown were propagated through the analysis. The data in Figure 4 are normalized to their sums over the measured angular range. This helps to highlight that in comparison to PFPE, the distribution from squalane is slightly less sharply directed. In contrast, that from squalene is more sharply directed and more strongly confined to the specular side than from PFPE. These trends are present at both delays, but the distributions from all three liquids become noticeably broader and more subspecular at the later delay, at least qualitatively consistent with the corresponding shifts in Figure 2 noted above.

To help provide a simple, single quantitative measure of the differences between the distributions in Figure 4, we show in Table 2 the proportion of the scattering within the measured angular range (−60° < θ_f_ < 60°) which falls to either side of the normal for each liquid at each delay. In all cases, the specular side (positive θ_f_) dominates but by substantially more for squalene (around 2.6:1 at the earlier delay) than for either PFPE or squalane (both around 1.8:1). The difference between squalene and the other two liquids is well beyond the uncertainties. The expected significant shifts away from specular scattering at the later delay are also reproduced, the ratios falling to around 1.8:1 for squalene, 1.5 for PFPE, and 1.4 for squalane.

**Table 2:** Percentage of Total OH Scattered with θ_i_ = 45° into θ_f_ to the Left of the Normal (i.e., Negative Angles, −60 to 0°) and to the Right of the Normal (Positive Angles, 0–60°)^a^

	132 μs		152 μs	
	–60 to 0°	0–60°	–60 to 0°	0–60°
PFPE	35.0 ± 0.7	65.0 ± 0.8	39.6 ± 1.0	60.4 ± 0.8
squalane	35.3 ± 0.7	64.7 ± 0.8	41.9 ± 1.1	58.1 ± 1.6
squalene	27.7 ± 1.6	72.3 ± 1.5	35.3 ± 0.7	64.7 ± 1.4

a Signals for θ_f_ = 0° were divided evenly between the two sides.

## Discussion

As noted in the Introduction, there was already strong evidence that the predominant OH scattering mechanism from all three liquids at these collision energies is IS-like. This is further reinforced here by the confirmation of the effect of incidence angle on the average most-probable scattered speeds in Table 1; as observed previously, the smaller deflection angles associated with θ_i_ = 45° correlate, as expected in an IS mechanism, with higher final speeds.^19−21^ Differences in final speeds between liquids are also clearly incompatible with TD-like behavior, with those in Table 1 consistent with previous observations and interpretations that PFPE is a “stiffer” surface than the hydrocarbon liquids squalane or squalene.^9−12^ The new observation here of the asymmetry in the angular distributions for θ_i_ = 45°, seen for the first time for squalane and squalene, is indisputable as further *prima facie* evidence of an IS mechanism. The less obvious and more interesting question is what might the differences in the angular distributions reveal about the interactions of OH with the different liquid surfaces?

We have interpreted the form of the angular distribution from PFPE in terms of surface roughness.^20^ As is well-known, in the absence of a significant contribution from the thermal motion of the surface, an atomically flat surface can only lead to *super*-specular (i.e., closer to the surface plane) scattering because any momentum transfer will be confined to the vertical component and will be from the projectile to the surface.^22^ The breadth of the distribution from PFPE was inferred to relate to some combination of the diversity of impact sites and the propensity for multiple deflections, which are both correlated with surface roughness and contribute to enhanced scattering of OH in less-specular directions.

It is therefore possible that the differences in the distributions for squalane and squalene relative to those of PFPE simply reflect differences in their surface structures. By implication from Figure 4, squalane would be marginally rougher than PFPE, with squalene being significantly smoother than either. However, we do not think that is the only possible or even the most likely explanation. We are not aware that it has yet been quantified in detail, but previous molecular dynamics (MD) simulations of the squalane and squalene surfaces do not suggest large differences in roughness.^17,23^ Their surface tensions, which would be expected to correlate with surface flatness, are also similar.^24−26^ Moreover, previous independent scattering of noble gases, including Ne which is kinematically most similar to OH, gave angular distributions which were, again, quite similar for PFPE and squalane; they were marginally broader and more subspecular from PFPE than from squalane (i.e., the opposite of any difference apparent in Figure 2 or 4 here).^27^ No similar noble-gas scattering data are available for squalene.

We suggest that an alternative explanation for the differences in the angular distributions may lie in which OH molecules survive a collision at the surface, i.e., that they are “filtered” by trajectory type in different ways.

For PFPE, previous data support the presumption, based on the absence of thermodynamically feasible reaction channels, that all the incident OH survives and is inelastically scattered. For squalane, the only feasible reaction is the abstraction of an H atom. As noted, measured survival probabilities imply that around 30% of the OH is lost overall. Being a direct process subject to a barrier, with heights that vary according to the different C–H bond types present in the molecule, it might seem at first sight that the reaction should be favored by collisions at higher kinetic energy.^28^ In this view, OH that is lost should preferentially come from trajectories which, in the absence of reaction, would correspond to “single-bounce” impulsive scattering. OH loss would be correspondingly less favored for trajectories where the OH survives an initial nonreactive encounter which dissipates some of its kinetic energy, also associated with a higher probability of scattering at more backward angles. There is perhaps some evidence for this in the angular distributions in Figure 2 and especially Figure 4 and the data in Table 2, with scattering from squalane slightly favoring more backward scattering than the purely inelastic scattering from PFPE. However, this argument may be oversimplified because those measurements that are available indicate that OH loss on squalane does not have a strong dependence on initial kinetic energy.^18^ This may be explained by the reduced ability to surmount the barrier on the first encounter being compensated by the larger number of opportunities to react in multiple-bounce trajectories; therefore, some caution should be exercised in interpreting small differences between squalane and PFPE.

For squalene, the situation is more clear-cut. The overall OH loss is higher at around 70%, at this collision energy. This alone does not necessarily indicate a change in mechanism, because the C–H bond sites in squalene are all allylic and have lower activation barriers to H-abstraction.^29^ However, we have demonstrated previously that OH loss *increases* markedly as the collision energy *decreases*, consistent with the negatively activated behavior seen for reactions of OH with smaller alkenes in the gas phase.^18^ We note that we can discount the enhanced loss of OH due to bulk solvation as a possible explanation because the absolute rates of reaction of OH with liquid hydrocarbons are too fast to allow the OH to survive long enough to diffuse to any significant depth. The proposed mechanistic interpretation was that the enhanced OH loss on squalene is the result of addition reactions at the unsaturated C=C sites, forming a nascent hydroxy-substituted radical. This was further supported by the relative absence of slower, rotationally colder scattered OH in earlier experiments in which photolytically generated, translationally and rotationally hot OH was scattered from squalene and squalane.^17,18^ This is also apparent here in the ratio of signals in Figure 3a(i),(ii) for squalane compared to those for squalene in Figure 3b(i),(ii). We believe that it is convincingly further corroborated by the OH angular distributions from squalene here. The reduction in scattering at more subspecular angles from squalene relative to PFPE in Figure 4 is consistent with predominantly single-bounce, impulsively scattered OH surviving, with those that have had their initial kinetic energy dissipated in the initial encounter being more likely to be lost by addition to a double-bond site on a subsequent encounter.

**Figure 5:**
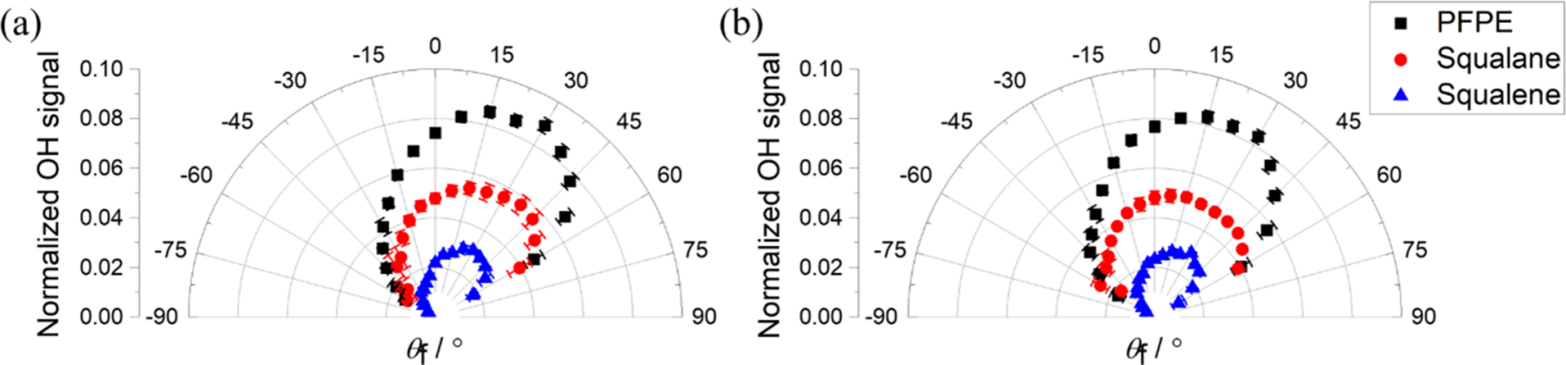
Angular distributions of OH scattered with θ_i_ = 45° from PFPE (black squares, from a reanalysis of data in ref (20)), squalane (red circles), and squalene (blue triangles) taken (a) 132 μs and (b) 152 μs after the creation of OH in the discharge. The distributions are derived from those in Figure 4 by weighting with the OH survival probability on each liquid, as measured in ref (18).

This possible interpretation is emphasized in the construction in Figure 5, where the distributions from the three liquids have been weighted by their previously measured relative survival probabilities.^18^ There is a significantly larger proportional loss from squalene relative to PFPE in more backward directions, associated with less direct trajectories, than in the specular region. In contrast, for squalane, the loss relative to PFPE is more evenly distributed but, if anything, higher in near-specular directions, consistent with the arguments above.

If correct, this signature of those (unobserved) molecules that reacted and were lost is an interesting new mechanistic diagnostic that we do not believe has been described previously for reactions at the gas–liquid interface, nor to our knowledge in gas–solid reactive scattering. We look forward to applying it, potentially in conjunction with more fully resolved scattered-speed distributions that carry related information, to reactions of OH with a wider range of liquid surfaces representing the diversity of functionalities present at atmospheric aerosol surfaces. It was already well understood that modeling of the progressive aging of the particles requires a knowledge of their current composition because of the variation of the OH uptake coefficient with exposed functional-group type. However, this work is also helping to reveal that the reaction mechanisms also differ fundamentally for different functional groups and therefore may also, for example, have very different temperature dependencies, which would not necessarily be well-described by conventional Arrhenius-like behavior.

## Conclusions

The principal new results here are the angular distributions for the inelastic scattering of OH from liquid squalane and squalene surfaces, which have been measured successfully for the first time. For non-normal incidence, the distributions are clearly asymmetric, reinforcing previous conclusions that the dynamics are predominantly impulsive at a collision energy of *E*_i_ = 35 kJ mol^–1^. There are subtle, but intriguing, differences between the distributions for these two liquids. We suggest that this may be explained by the differing trajectory types that lead to OH surviving and scattering inelastically. The results are consistent with less direct trajectories preferentially *escaping* from squalane, for which H-abstraction is the only possible reaction path. In contrast, on squalene, for which there is also a radical-addition pathway, less direct trajectories appear to be preferentially *lost*.

## Supporting Information Available

Removing background squalane fluorescence from experimental images; most-probable scattered speeds as a function of incidence and scattering angles; flux-density (FD) correction; weighting the *N′*-averaged OH scattered angular distributions by the rotational populations; FB corrections for extended images vs image sequences*;* and *N′*-dependent angular distributions of OH scattered from PFPE, squalane, and squalene (PDF)
