# Supplementary material for: Distinguishing Mechanisms for Reactive Uptake at Liquid Surfaces via Angular Distributions of Inelastically Scattered Molecules
Source: J Phys Chem A. 2024 Jun 25;128(26):5166–74. doi: 10.1021/acs.jpca.4c02917 (PMC11229065; doi:10.1021/acs.jpca.4c02917)
Supplement: Supplementary file 1 — jp4c02917_si_001.pdf [file jp4c02917_si_001.pdf]

Distinguishing Mechanisms for Reactive Uptake at Liquid Surfaces *via* Angular Distributions of Inelastically Scattered Molecules

*Maksymilian J. Roman, Adam G. Knight, Daniel R. Moon, Paul D. Lane, Matthew L. Costen, and Kenneth G. McKendrick**

Institute of Chemical Sciences, Heriot-Watt University, Edinburgh EH14 4AS, UK

Supplementary Material

Table of contents

Contents

S1: Removing background squalane fluorescence from experimental images. S2

S2: Most-probable scattered speeds as a function of incidence and scattering angles. S3

S3: Flux-density (FD) correction S4

S4: Weighting the *N*′-averaged OH scattered angular distributions by the rotational populations. S6

S5: FB (finite-beam) corrections for extended images vs. image sequences S7

S6: *N*′-dependent angular distributions of OH scattered from PFPE, squalane, and squalene. S8

***Contact details:** k.g.mckendrick@hw.ac.uk

S1: Removing background squalane fluorescence from experimental images.

When taking the experimental extended images of OH scattered from squalane, an additional fluorescence signal was discovered, which was excited by stray light from the probe laser sheet. The origin of this background fluorescence is most likely impurities in the squalane liquid, although their precise character is currently unknown. No similar signals were seen for squalene or PFPE.

The background fluorescence is outlined approximately (in red) in Figure S1. The signal overlapped precisely with the image of the rotating wheel as seen by the camera. The wheel appears as a narrow ellipse from this perspective because the central axis of the imaging system is displaced (in the vertical direction in Fig. S1) by approximately 25 mm from the surface of the wheel. Note that this perspective effect does not cause any significant blurring of the pLIF image because the laser sheet which excites it has a narrow depth perpendicular to the plane of the figure. The near edge of the laser sheet is ~10 mm from the closest point of the wheel; the extrapolated sheet would inscribe a chord on the wheel ~15 mm above its rotation axis and ~10 mm below its top edge (in the lab frame). This intersects closely with the brightest part of the background fluorescence signal.

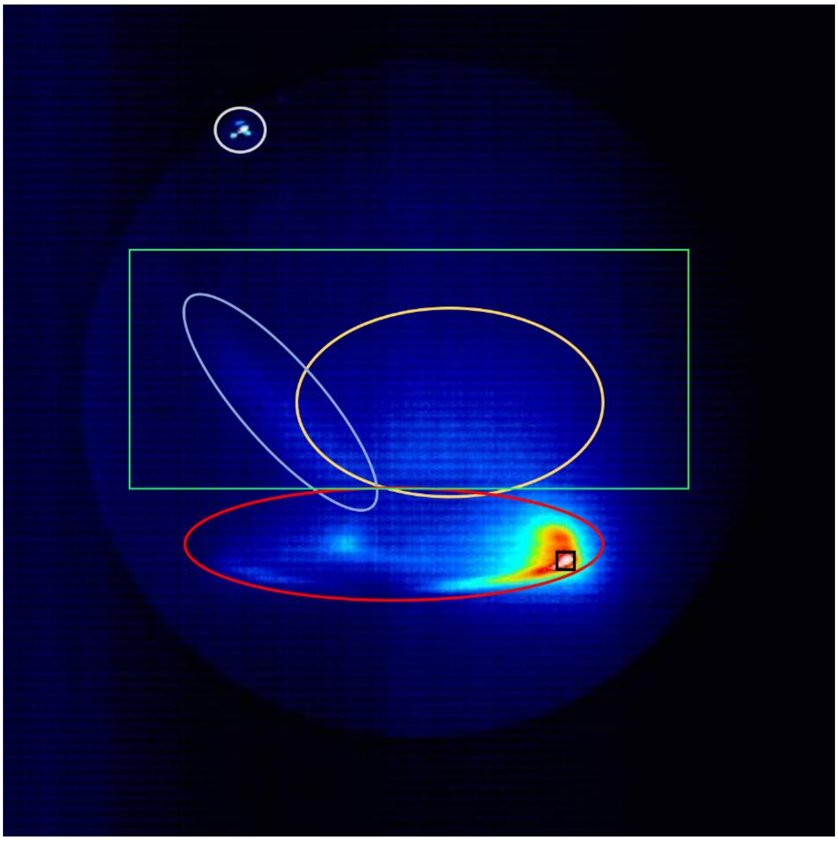

Figure S1: A raw (not corrected for the instrument function) extended image of OH (N' = 3) scattered from the surface of squalane at a discharge-probe delay of 132 μs. The approximate region irradiated by the laser sheet and from which pLIF signals can be collected is shown as a green rectangle (compare Figure 3 of the main text). All sources of pixel intensities are outlined: residual ingoing OH beam at θ_i = 45° (pale blue), scattered OH (yellow), squalane fluorescence (red). The localised signal (outlined in pale grey) comes from damaged MCP channels. The black square indicates the ROI used in the subtraction process described in this section.

As can be seen in Fig. S1, the background fluorescence signal only marginally overlaps the region (indicated approximately by the green rectangle) containing genuine OH pLIF signals. Nonetheless, it was eliminated by subtracting images taken with no OH present in the chamber ("squalane-only images"), but otherwise with the same conditions as for the experimental images (*i.e.*, pLIF wavelength and discharge-probe delay, etc.). To ensure the squalane signal was subtracted precisely, the squalane-only images were scaled so that the summed pixel intensities within a small square ROI (marked in black on Figure S1) were the same in an experimental image and its equivalent squalane-only image.

S2: Most-probable scattered speeds as a function of incidence and scattering angles.

The final-angle-dependent scattered OH most probable speeds were measured from the experimental image sequences in the same manner as those for OH scattered from PFPE described previously.[1] These speeds are tabulated for OH (*N′* = 2, 3, and 4) scattered from squalane (Table S1) and squalene (Table S2) for two incidence angles, θ_i = 0° and 45°. Note that no reliable speeds could be measured at a few scattering angles because of the ROIs proximity to the edge of the probe region (*i.e.*, laser sheet edge) and/or overlap with the ingoing beam.

Table S1: Most probable speeds (in m s^{-1}), with 1σ uncertainties, of OH scattered from squalane.

θ_f / °	θ_i = 0°			θ_i = 45°		
	N′ = 2	*N′* = 3	*N′* = 4	*N′* = 2	*N′* = 3	*N′* = 4
-60	1072 ± 250	1351 ± 65	1460 ± 314	-	-	-
-45	1018 ± 53	1166 ± 62	1294 ± 181	1246 ± 182	1276 ± 97	1388 ± 132
-30	1054 ± 24	1134 ± 26	1106 ± 23	1130 ± 69	1059 ± 41	1434 ± 166
-15	1086 ± 11	1159 ± 69	1079 ± 41	1204 ± 56	1223 ± 90	1084 ± 68
0	-	1042 ± 81	1150 ± 64	1164 ± 81	1370 ± 86	1479 ± 193
15	1251 ± 36	1140 ± 63	1087 ± 55	1305 ± 27	1316 ± 76	1531 ± 223
30	1129 ± 19	1126 ± 22	1180 ± 24	1350 ± 41	1406 ± 49	1439 ± 62
45	1102 ± 95	1284 ± 73	1217 ± 78	1466 ± 84	1470 ± 106	1718 ± 153
60	-	-	1171 ± 38	1387 ± 143	-	-

Table S2: Most probable speeds (in m s^{-1}), with 1σ uncertainties, of OH scattered from squalene.

θ_f / °	θ_i = 0°			θ_i = 45°		
	N′ = 2	*N′* = 3	*N′* = 4	*N′* = 2	*N′* = 3	*N′* = 4
-60	1172 ± 161	1011 ± 199	-	-	-	-
-45	1535 ± 34	992 ± 65	1402 ± 164	-	-	-
-30	1350 ± 79	1012 ± 31	1257 ± 32	-	1036 ± 97	-
-15	-	1006 ± 50	1304 ± 100	1335 ± 175	1122 ± 156	1282 ± 154
0	-	1013 ± 39	1372 ± 110	1378 ± 100	1227 ± 38	1383 ± 65
15	-	1022 ± 40	1299 ± 140	1391 ± 41	1137 ± 145	1430 ± 60
30	1351 ± 80	968 ± 112	1273 ± 132	1382 ± 75	1313 ± 67	1390 ± 78
45	1318 ± 105	1144 ± 125	1413 ± 9	1311 ± 110	1738 ± 48	1308 ± 68
60	1406 ± 528	-	-	1081 ± 111	-	-

S3: Flux-density (FD) correction

The FD correction accounts for differences in scattered speeds as a function of θ_f, allowing relative fluxes at different angles to be determined from the measured relative number densities. It corrects for the very well-known effect that, for the same flux, slower-moving molecules will spend longer in the observation zone and hence be relatively over-detected by a number-density detector such as LIF, and vice versa for faster molecules.

The FD correction is negligible for θ_i = 0°, because the measured values of peak speed are effectively independent of θ_f, within the uncertainties, for all three liquids (see Section S2 for squalane and squalene, and similar previous measurements for PFPE[1]).

This is no longer the case for θ_i = 45°, for which there is discernible a general increase in peak speed from the most-backward to the most-forward angles. This is as expected for impulsive scattering, where less momentum has been transferred to the surface for smaller deflection angles.

Of the several measurements that we have of final speeds for θ_i = 45° with different liquids, those with the best signal-to-noise and hence lowest statistical errors are from our original proof-of-concept work.[2] Those absolute speeds are now known to have been systematically underestimated, based on a recalibration of the absolute length scale and as confirmed by the higher speeds in the more recent measurements.[1] We therefore used the corrected absolute values of the previous relative speeds as the best-estimate input to the Monte Carlo modelling of flux-density effects. In practice, although there is no *a priori* reason why they should be strictly linear, the peak speeds as a function of θ_f are well-fit by a straight line across the measured range for all three liquids. The resulting forms of the most-probable speeds for each liquid used in the modelling are shown in Figure S2.

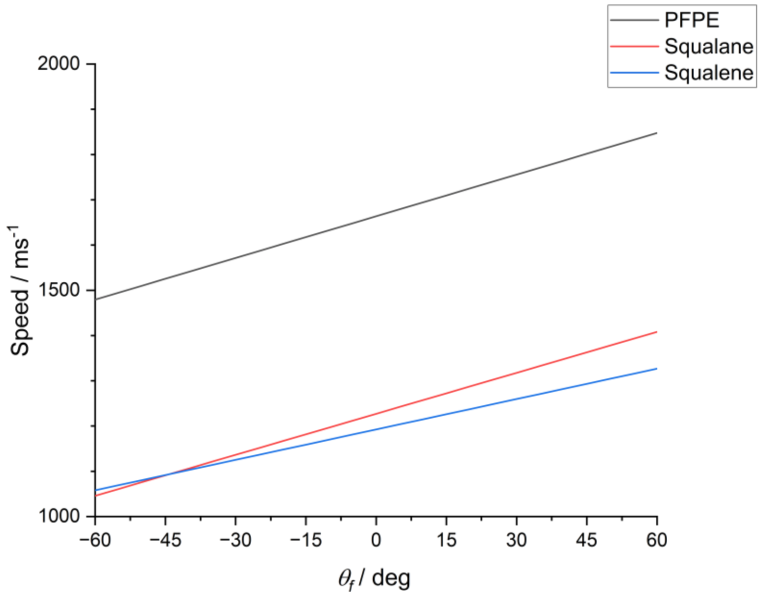

Figure S2: Assumed speeds as a function of final angle for θ_i = 45°, used as input in MC modelling of flux-density effects. OH scattering from PFPE (black), squalane (red), squalene (blue).

Additional MC modelling was then carried out based on these final-speed distributions, along with parameters that best-described the realistic input molecular beam and other geometric factors in

the real measurements.[3] To avoid possible artefacts from an unnaturally sharp distribution of final speeds at each θ_f, which we know is not a good description of the experimental distributions based on the measured TOF profiles, an additional Gaussian width was superimposed on the scattered speeds at each angle. This width was set to 200 ms^{-1}, based on the approximate width of the main peaks in the TOF distributions. In practice, the specific choice of this width was found not to materially affect the results.

In essence, the FD correction factors were determined by dividing the scattered flux at each angle assumed as input in the MC simulation by the output number densities predicted by the model at that angle. The predicted number densities were subjected to the same ROI-based analysis as the experimental data. Since we do not have sufficient information to parameterise the full scattered speed distribution (not just peak speeds) as a function of angle reliably, the outcome is effectively the same regardless of whether the predicted TOF profiles for selected ROIs are integrated between fixed limits (as in the analysis of image sequences) or a snapshot of number densities is taken near the peak delay (as in extended images).

The FD factors so derived can then be applied as multiplicative factors to the real measurements of relative number densities to convert them to relative fluxes. Figure S3 shows the results. To guide the eye, the green line shows the (hypothetical) absence of any variation in the FD factor with θ_f. For all three liquids, the real FD factors show the expected left-right distortion. It is in the sense anticipated above, because the molecules scattered to more-positive θ_f have higher speeds (see Fig. S2) and hence lower number densities for the same flux, requiring a larger FD correction factor. The FD factors range from ~+10% at θ_f = +60° to ~-10% at θ_f = -60°, relative to scattering along the normal. This correction is non-negligible and has been applied to the angular distributions from all three liquids reported in Figures 2, 4 and 5 in the main text. This includes a re-analysis of the PFPE data, for which this factor had not previously been considered.[1] In effect, this means that the true flux distributions are slightly more left-right asymmetric than is already apparent in the raw measurements of number densities.

Significantly, though, as noted in the main text, the differences between the FD factors for scattering from different liquids are very marginal. This reflects the similar slopes in Figure S2. An important implication is that any differences in the measured angular distributions from different liquids cannot be explained as being simply due to differential flux-density effects.

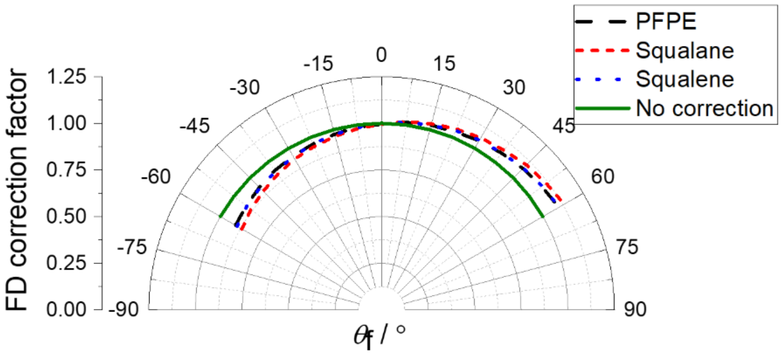

Figure S3: Comparison between θ_f-dependent FD correction factors for OH scattered from PFPE (black long dashed), squalane (red short dashed), and squalene (blue dotted) surfaces. The green solid line portrays a uniform “no correction” value and is included for comparison purposes.

S4: Weighting the *N*′-averaged OH scattered angular distributions by the rotational populations.

The angular distributions of scattered OH determined from the image sequences (reported in Fig. 2 of the main text) or extended images (Fig. 4 of the main text) are averages over the measured *N*′ rotational levels. This averaging was done by weighting contributions from each *N*′ by the relative population of that level. In this work, we were only able to qualitatively estimate the relative *N*′-populations from the relative intensities of experimental images and no quantitative new analysis of the rotational population distributions was performed. However, previous experiments on OD scattering from squalane and squalene included such measurements, which provided us with reliable estimates on which to base the weighting. The population distributions themselves obviously cannot be compared directly, but we accounted for the differences in rotational-level spacings in OH and OD by assuming they were described by equivalent rotational temperatures. Table S3 shows the relative populations of *N*′ rotational level used in the averaging. The temperatures extracted from fits to the distributions for different liquids overlap within their uncertainties, so the weightings are essentially the same for all three liquids at this level of precision.

As noted in the main text, we are detecting on Q_1 branches, which probe one of the Λ- doublets in the majority F_1 manifold, to characterise the distributions. We believe it very unlikely, for dynamical reasons, that there would be differences in angular distributions between Λ- doublets. We also do not expect significant differences in those for the minority F_2 manifold. Within the F_1 manifold, it is possible that the final weighted angular distributions may change if the data for N' = 1 were to be collected. However, we do not expect this to have a substantial effect on the final OH scattered angular distributions. The contribution from N' = 1 at the relevant rotational temperatures (around 400 K) is comparable to that from N' = 3, and only around 27% of the total F_1 population in N' = 1-4. There is also no reason to think the N' = 1 distribution will be very different from that of the other levels. Similarly, at these temperatures, only around 18% of the F_1 population is in all the levels with $N' \geq 5$. We therefore consider it unlikely that different angular distributions within this minor, but similar-sized, component of unobserved higher levels would perturb the overall angular distributions from different liquids significantly.

Table S3: Relative populations of *N*′ rotational levels of scattered OH.

	N' = 2	N' = 3	N' = 4
Population	0.44	0.39	0.17

S5: FB (finite-beam) corrections for extended images vs. image sequences

The FB correction factors were originally derived[3] by integrating the TOF number-density profile for a given RoI, equivalent to the way that the image sequences were analysed to produce flux angular distributions. We have confirmed that these factors were also applicable to the subtly different situation in the image sequences, where the measured number densities are a snapshot at a given delay. This was done by carrying out an MC simulation using the parameters best-matching the characteristics of the realistic molecular beam and other experimental variables, and analysing the predicted number density distributions using the same procedures as for the experimental image sequences and extended images, respectively. The resulting FB factors are shown in Figure S4, where it can be seen that there are only marginal differences between the results for the image-sequence approach and the extended-image approach at the peak of the scattered signal.

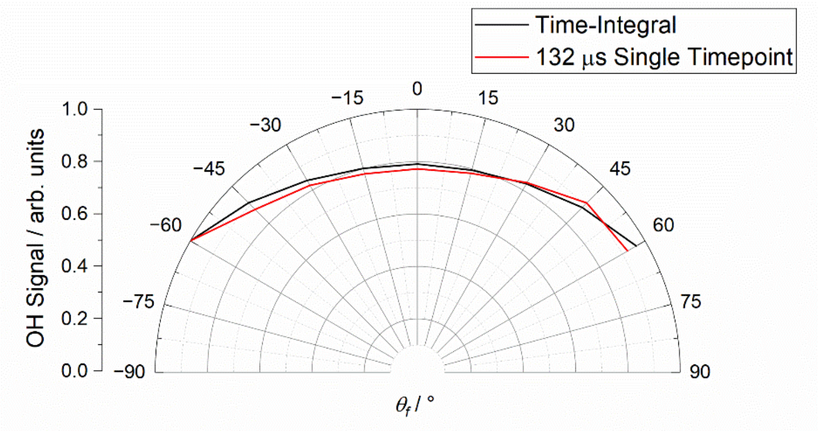

Figure S4: Comparison between θ_f-dependent FB correction factors. Predicted number densities processed as in image sequences (black) or extended images at the peak delay of 132 μs (red).

S6: *N*′-dependent angular distributions of OH scattered from PFPE, squalane, and squalene.

Figures S5-S7 show the angular distributions of OH in rotational levels N' = 2, 3, and 4 scattered with incidence angle θ_i = 45° from surfaces of PFPE (Fig. S5), squalane (Fig. S6), and squalene (Fig. S7). In each case, the distributions are measured at discharge-probe delays of 132 and 152 µs. Distributions are shown in sequence of the corrections mentioned in the main text; (a) number-density distributions extracted directly from IF-corrected extended images, (b) number-density distributions adjusted by the FB correction, and (c) flux distributions adjusted by both the FB and FD corrections.

Note that the effect of the FB correction in each case is to compress the distribution laterally and hence shift the peak towards more-normal scattering; this is as expected, to counteract the finite spread of the incident MB on the observed distribution. As we have quantified previously through MC simulations using a realistic representation of the experimental parameters, for θ_i = 45° the correction is slightly asymmetric but results in a ~20% enhancement along the normal relative to θ_f = -60° and ~15% relative to θ_f = +60°.[3]

In contrast, the FD correction increases the left-right asymmetry and shifts the peak back towards more-specular angles; this is also as expected, as explained in Section S3, because of the correlations between scattered speed and θ_f. As stated there, the magnitude of the FD factors range from ~+10% at θ_f = +60° to ~-10% at θ_f = -60°, relative to scattering along the normal.

Consequently, the two effects partially cancel, as can be seen by inspection in Figures S5-S7.

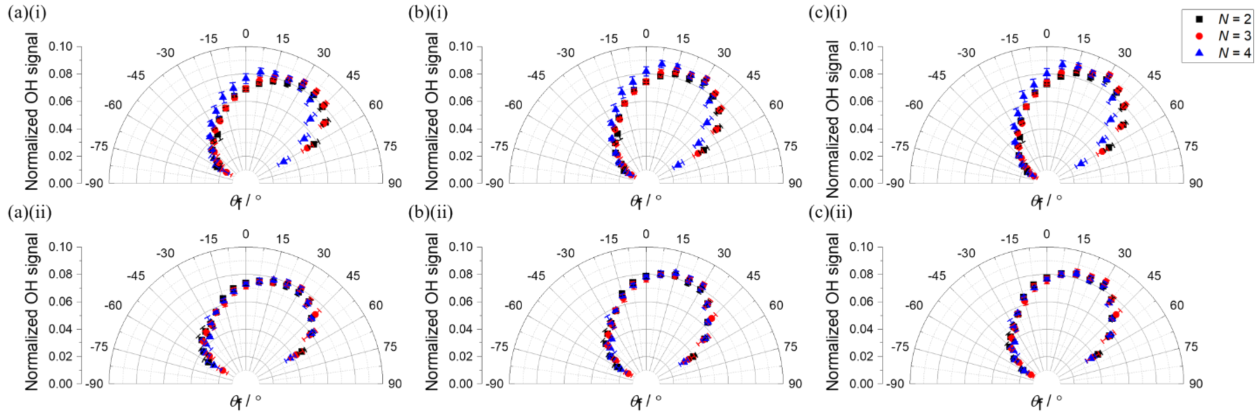

Figure S5: Sum-normalized angular distributions of OH in N = 2 (black squares), 3 (red circles), and 4 (blue triangles), scattered from PFPE at delays of (i) 132 µs and (ii) 152 µs after the HV discharge. Data represent: (a) integrated number-density distribution in specific ROIs, corrected only for the instrument-function (IF); (b) number-density distribution with additional finite molecular-beam (FB) correction, and (c) flux distribution, following both the FB and the flux-density (FD) corrections, as described in the main text. Results are averages over the three innermost arcs of ROIs; error bars are standard errors coming from the averaging process.

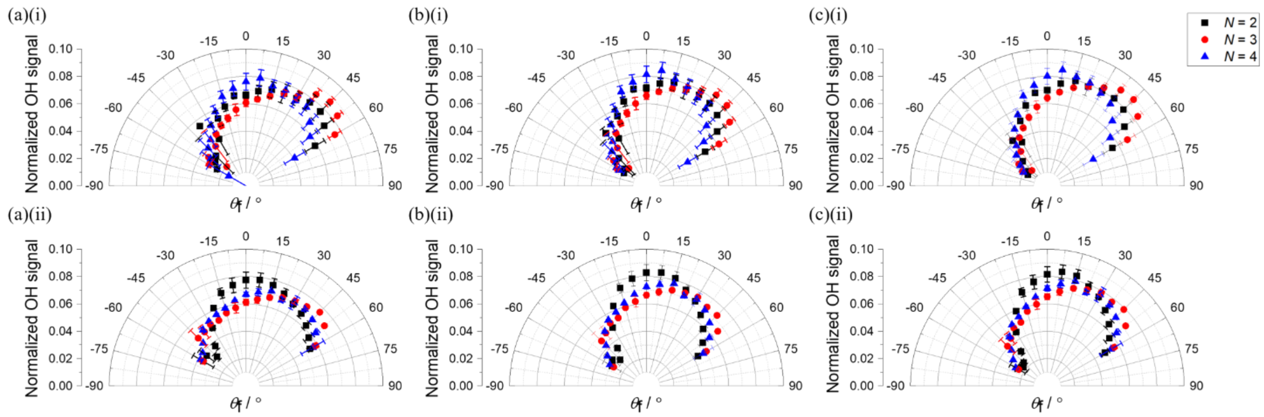

Figure S6: Sum-normalized angular distributions of OH scattered from squalane. All other labels and conditions as in Fig. S5.

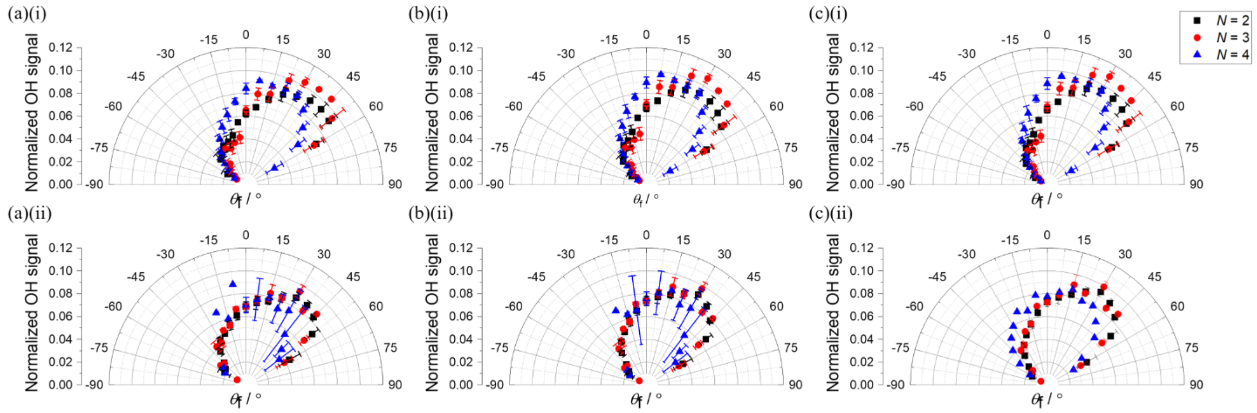

Figure S7: Sum-normalized angular distributions of OH scattered from squalene. All other labels and conditions as in Fig. S5.

References for Supplementary Information

(1) Roman, M. J.; Knight, A. G.; Moon, D. R.; Lane, P. D.; Greaves, S. J.; Costen, M. L.; McKendrick, K. G. Inelastic Scattering of OH from a Liquid PFPE Surface: Resolution of Correlated Speed and Angular Distributions. *J. Chem. Phys* **2023**. https://doi.org/10.1063/5.0153314.

(2) Bianchini, R. H.; Roman, M. J.; Costen, M. L.; McKendrick, K. G. Real-Space Laser-Induced Fluorescence Imaging Applied to Gas-Liquid Interfacial Scattering. *J. Chem. Phys.* **2019**, *151* (5), 054201. https://doi.org/10.1063/1.5110517.

(3) Knight, A. G.; Sieira-Olivares, C.; Roman, M. J.; Moon, D. R.; Lane, P. D.; Costen, M. L.; McKendrick, K. G. Quantifying the Dynamical Information Content of Pulsed, Planar Laser-Induced Fluorescence Measurements. *J. Chem. Phys* **2023**. https://doi.org/10.1063/5.0153321.
